# Transcriptional regulatory network analysis identifies *GRN* as a key regulator bridging chemotherapy and immunotherapy response in small cell lung cancer

**DOI:** 10.1186/s13045-025-01667-5

**Published:** 2025-02-05

**Authors:** Seungyeul Yoo, Ayushi S. Patel, Sarah Karam, Yi Zhong, Li Wang, Feng Jiang, Ranran Kong, Sharon Bikvan, Wenhui Wang, Abhilasha Sinha, Charles A. Powell, Jun Zhu, Hideo Watanabe

**Affiliations:** 1 GeneDx, 333 Ludlow Street, North Tower, 6th Floor, Stamford, CT 06902 USA; 2 Division of Pulmonary, Critical Care and Sleep Medicine, Department of Medicine, Icahn School of Medicine at Mount Sinai, 1468 Madison Avenue, New York, NY 10029 USA; 3 Tisch Cancer Institute, Icahn School of Medicine at Mount Sinai, 1425 Madison Avenue, New York, NY 10029 USA; 4 Department of Thoracic Surgery, The Second Affiliated Hospital of Xi’an Jiaotong University, Xi’an, China; 5 Department of Genetics and Genomic Sciences, Icahn School of Medicine at Mount Sinai, 1468 Madison Avenue, New York, NY 10029 USA

## Abstract

Small cell lung cancer (SCLC) is an aggressive and heterogeneous subtype, representing 15% of lung cancer cases. Although SCLC initially responds to etoposide and platinum (EP) chemotherapy, nearly all patients relapse with resistant tumors. While recent advances in immunotherapy have shown promise, only 10–20% of patients benefit, and effective stratification methods are lacking. The mechanisms of resistance to both therapeutics remain obscure. In this study, we aimed to gain insights into those leveraging a recent surge in the field of SCLC genomics. We constructed a regulatory network for SCLC and identified granulin precursor (*GRN*) as a hub of EP response associated genes. *GRN*-low patients showed improved survival with chemotherapy, while *GRN*-high patients exhibited resistance. GRN overexpression in SCLC cells conferred resistance to EP treatment and suppressed neuroendocrine features. *GRN* and its associated genes were linked to cancer cell intrinsic immunogenicity, and single-cell RNA-seq data revealed that *GRN* expression is particularly high in subsets of tumor-associated macrophages. In concordance with these findings, *GRN*-low tumors showed significantly better survival with chemo-immunotherapy, while *GRN*-high tumors did not benefit from additional immunotherapy. *GRN*-high tumors, associated with non-neuroendocrine (non-NE) subtypes, had a higher level of macrophage infiltration, potentially contributing to immunotherapy resistance. These results highlight GRN as a critical regulator of chemo-resistance and a potential biomarker for immunotherapy resistance in SCLC. Targeted therapeutic strategies for GRN-low patients could improve outcomes, while new approaches are needed for GRN-high patients. Overall, our findings implicate GRN as a bridge between chemotherapy and immunotherapy resistance through GRN-mediated mechanisms.

**Supplementary Information:**

The online version contains supplementary material available at 10.1186/s13045-025-01667-5.

To the editor

Small cell lung cancer (SCLC) is the most aggressive form of lung cancer, with most patients developing rapid resistance to etoposide and platinum (EP) chemotherapy, leading to poor outcomes [1]. While immune checkpoint blockade (ICB) therapies have demonstrated promise, only a minority of patients (10–20%) benefit [2], and the molecular mechanisms behind therapeutic resistance are still not well understood. In this study, we performed a comprehensive regulatory network analysis and identified granulin precursor (GRN) as a critical regulator associated with resistance to both chemotherapy and immunotherapy. Here, we summarize the key findings from our investigation, highlighting the functional role of GRN and its potential as a biomarker for stratifying patients based on their likelihood of benefiting from these therapies.

## *GRN* is a key regulator of chemo-resistant genes in SCLC

**Fig. 1:**
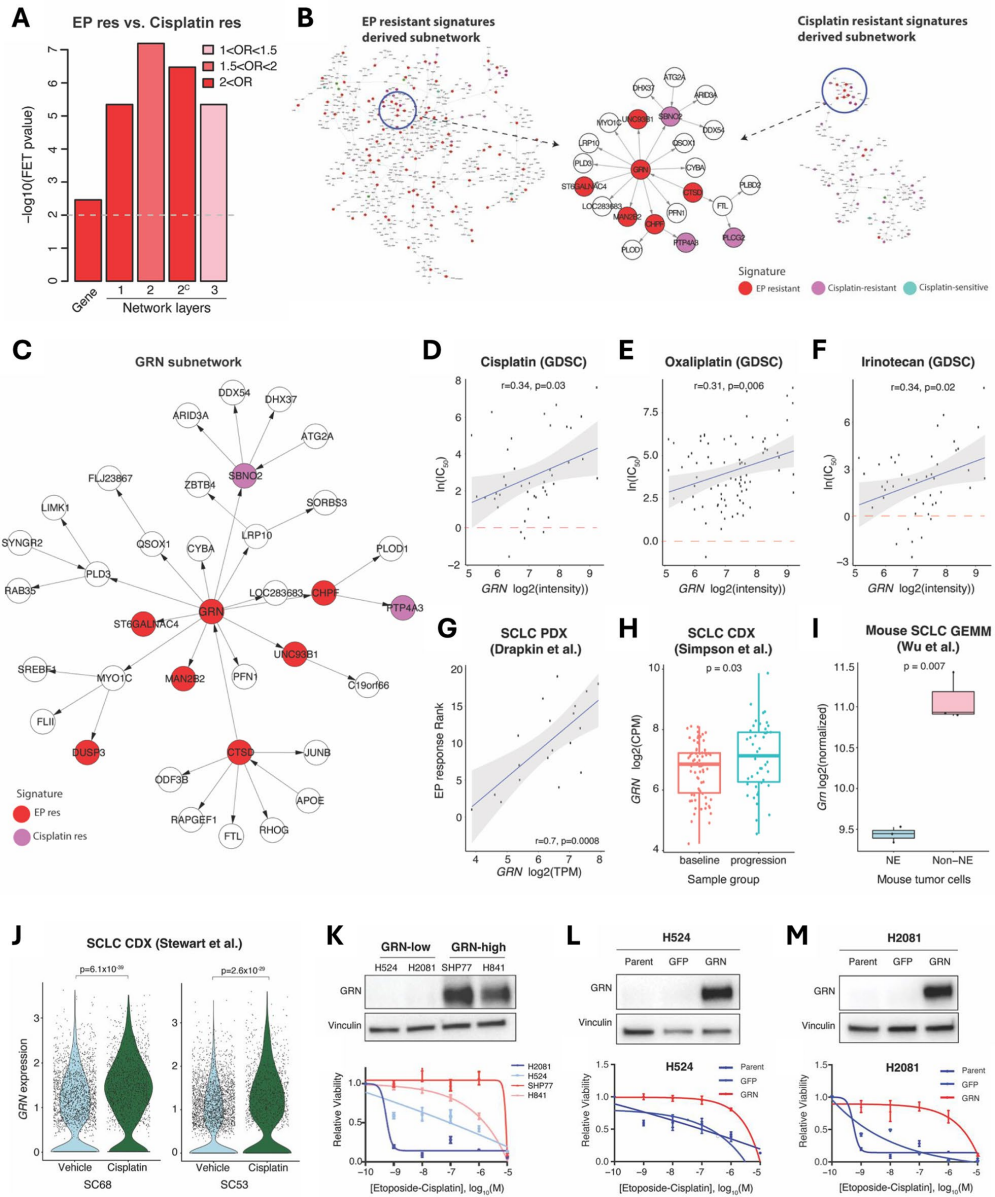
A causal regulatory network of small cell lung cancer identifies GRN as a key regulator of chemo-resistant signatures. **A** Overlap of network neighbors of chemo-resistance signature genes. EP resistant signatures derived from a PDX model (EP res) and cisplatin resistant signatures derived from a mouse model (Cisplatin res) were projected on the network with the signatures as seed nodes and their n-layer neighbors in the network were collected Gene: direct overlap of signature genes without network expansion, 1: overlap of one-layer neighboring nodes from seeds, 2: overlap of two-layer neighboring nodes from seeds, 2^c^: overlap of the largest connected subnetwork among the two-layer neighboring nodes, 3: overlap of three-layer neighboring nodes from seeds. The bin colors indicate odd ratio (OR) of the overlap between EP res and Cisplatin res associated gene sets. Fisher’s exact test (FET) p-values were -log10 transformed. **B** The largest connected subnetworks of the two-layer neighboring nodes of Ep res (left) and Cisplatin res (right). The intersected subnetwork is highlighted (middle). **C** A subnetwork within two layers from *GRN*. EP res and cisplatin res genes are colored in red and purple, respectively. **D**–**F** Correlation between *GRN* expression in SCLC cell lines (x-axis) and IC_50_ values (y-axis) to SCLC drugs (cisplatin, oxaliplatin, and irinotecan) downloaded from GDSC database [6]. Ln(IC_50_) zero value corresponding to 1 µM is marked in red dashed line. **G** Correlation between *GRN* expression (x-axis) and the ranking of EP response (y-axis) in 19 SCLC PDX models [3]. **D**–**G** Pearson correlation coefficients and p-values are calculated. **H** *GRN* expression (y-axis) difference between baseline and progressed tumors from SCLC CDX [7]. **I** *Grn* expression (y-axis) difference between neuroendocrine and non-NE SCLC cells from *Rb1*^f/f^;*Trp53*^f/f^; *Pten*^f/f^ TKO GEMM [8]. **H**–**I** Two-sided t-test p-value is calculated to assess the differences. **J** *GRN* expression (y-axis) between vehicle- and cisplatin-treated CDX models from patients SC68 and SC53 [9], respectively. Expression differences are assessed by Wilcoxon rank-sum test. **K** (Above) Western blots showing protein expression of GRN in four SCLC cell lines. (Below) Viability of each cell lines with equimolar treatment of EP for 96-h relative to vehicle control. IC_50_ =  ~ 18.83uM and ~ 1.86uM for SHP77 and H841, and ~ 55 nM and ~ 0.54 nM for H524 and H2081, respectively. **L**, **M** (Above) Western blots confirming GRN over-expression (OE) in the two GRN^low^ cells (H524 and H2081). (Below) Viability of each cell line with equimolar treatment of EP for 96-h relative to vehicle control. IC_50_ > 1uM for both H524 and H2081 with GRN OE

To identify consistent transcriptomic features underlying chemo-resistance in SCLC, we analyzed 359 EP-resistant genes (*EP-res*) from SCLC patient-derived xenografts (PDX) [3] and 223 cisplatin-resistant genes (*Cisplatin-res*) from genetically engineered mouse models (GEMMs) [4] (Table. S1). While the overlap was statistically significant between two signatures (Fisher Exact Test (FET) Odd-ratio (OR) = 2.9 and *p* value = 0.005), small number of overlapping genes (n = 9) limited further investigations on common molecular mechanisms (Fig. 1A). By constructing a molecular causal network from 135 SCLC primary tumors using Bayesian Network (BN) approach [5] (Fig. S1A), we identified a two-layer subnetwork that included 43 shared genes, demonstrating the utility of network analysis in connecting both signatures (Fig. 1A). Key driver analysis revealed *GRN* as the central hub gene for chemo-resistance connecting both signatures (Fig. 1B, C, Table S2).

The role of *GRN* as a chemo-resistance driver was further confirmed across multiple independent SCLC transcriptomic data from SCLC cell lines [6], PDX [3], Circulating Tumor Cells derived xenograft (CDX) [7], and GEMM models [8], where *GRN* expression was significantly associated with chemotherapy responses (Fig. 1D–I, Fig. S1B–G). Notably, in paired CDX samples from two patients, that were initially sensitive to cisplatin but relapsed after treatment [9], *GRN* expression was significantly higher in cisplatin-treated samples (Fig. 1J). We showed that SCLC cell lines overexpressing GRN exhibited increased resistance to EP, with significantly higher IC50 values compared to GRN-low cells (Fig. 1K). While GRN knockdown did not restore chemo-sensitivity in resistant cells (Fig. S1H), GRN overexpression in sensitive cells was sufficient to induce resistance (Fig. 1L-M), suggesting GRN’s critical role in initiating, but not maintaining, a chemo-resistant state.

## *GRN* and its associated genes classify SCLC patients into groups with distinct therapeutic responses

**Fig. 2:**
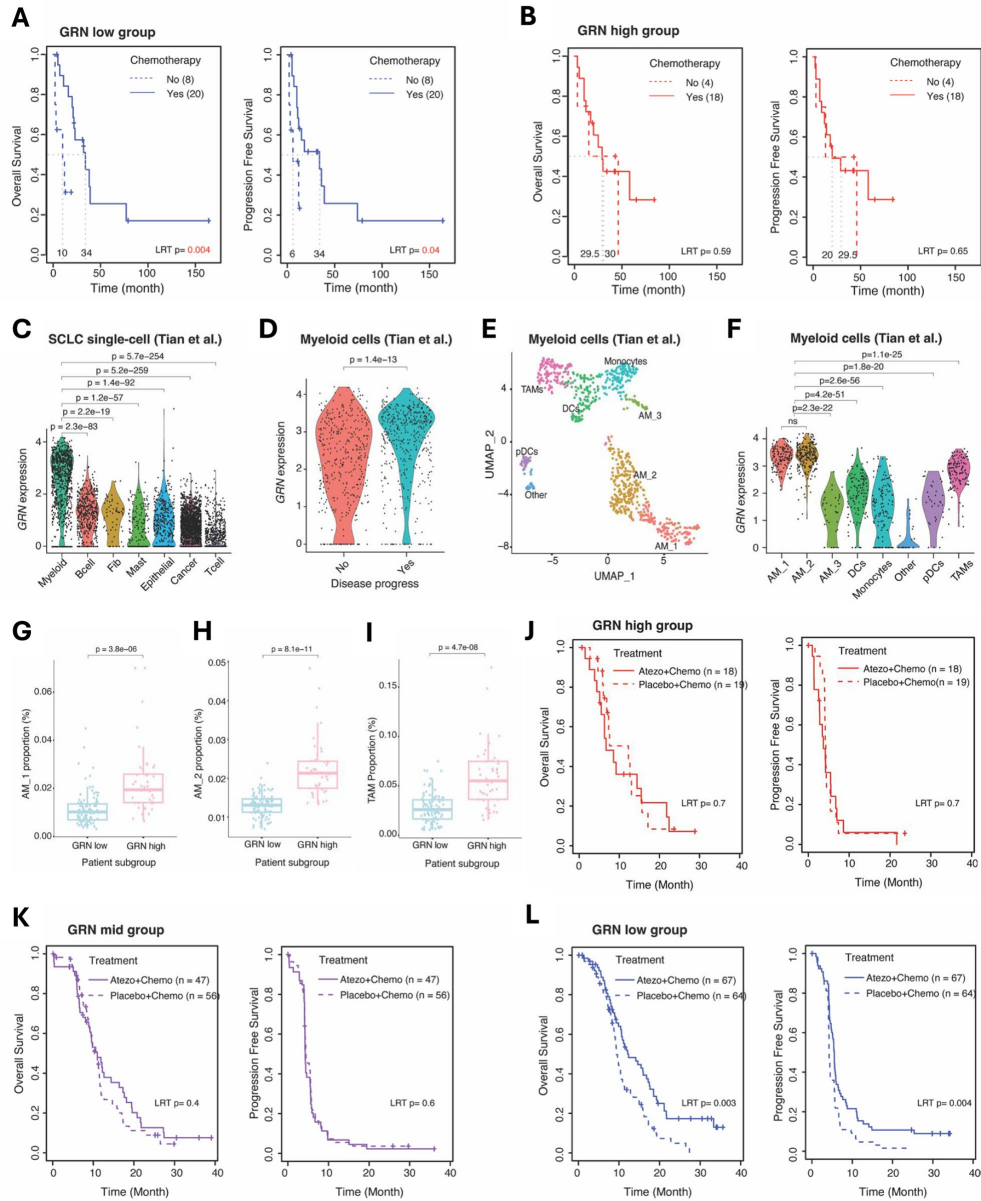
*GRN* and Its Associated Genes Classify SCLC Patients into Groups with Distinct Therapeutic Responses. **A**, **B** KM plots (left: OS and right: PFS) showing survival differences between chemo-treated and not-treated patients in *GRN*-low group (**A**) and in *GRN*-high group (**B**). Samples from George et al. whose survival information available are included in the analysis. Log rank test (LRT) p-values are calculated to assess survival differences between the groups. **C** *GRN* expression (y-axis) in various cell types in SCLC primary tumors. Cell type annotation information is from the original paper [10]. **D** *GRN* expression (y-axis) varies in myeloid cells from patients with different disease progression. **E** UMAP plot showing multiple myeloid subtypes annotated in the original paper [10]. **F** *GRN* expression (y-axis) in myeloid subtypes. Expression differences are assessed by Wilcoxon rank-sum test. **G**–**I** Cell fractions are estimated in bulk tissue expression profiles of primary tumors for alveolar macrophages 1/2, and tumor-associated macrophages in *GRN*-high and *GRN*-low tumors classified based on *GRN*-associated genes. Two-sided t-test p-values are assessed for cell fraction differences. **J**–**L** KM plots showing survival differences between Atezolizumab and placebo in *GRN*-high, in *GRN*-mid, and in *GRN*-high groups for and overall and progression-free survival respectively. Log rank test (LRT) *p* values are calculated to assess survival differences between the groups

We then identified GRN-associated genes, 249 GRN^+^ and 124 GRN^−^genes (Table S3), using CCLE data to evaluate how GRN mediates tumor-intrinsic resistance, which are also associated with both primary and acquired chemo-resistance (Fig. S2). When we classified human SCLC tumors by the GRN-associated genes into *GRN*-high (n = 45) and *GRN*-low (n = 90) groups (Fig. S3), *GRN*-low group showed better survival in both Overall Survival (OS) and progression free survival (PFS) with chemotherapy (Fig. 2A), while *GRN*-high group did not differ in survival with or without chemotherapy (Fig. 2B).

Single-cell RNA-seq analysis of human SCLC tumors [10] revealed that *GRN* was highly expressed in alveolar macrophages (AMs) and tumor-associated macrophages (TAMs), especially in patients with progressed disease (Fig. 2C–F, Fig. S4). Cellular composition of the bulk SCLC tumors by deconvolution [11] revealed a significant positive correlation between *GRN* expression and the proportion of macrophages, particularly AM1, AM2, and TAMs (Fig. 2G–I). This suggests that *GRN*-high tumors have a more macrophage-enriched TME, which may contribute to both chemo-resistance and immunotherapy resistance.

Given these results, we explored whether *GRN* could serve as a biomarker for predicting immunotherapy response. Analysis of 271 treatment-naive patients from the IMpower133 clinical trial data [12], which combined chemotherapy with ICB (anti-PD-L1), showed that patients with *GRN*-high and *GRN*-mid tumors did not benefit from additional immunotherapy (Fig. 2J, K). In contrast, patients with *GRN*-low tumors showed a significant survival benefit from the combination of chemotherapy and immunotherapy (Fig. 2L). This differential response supports the potential of *GRN* as a stratification biomarker for ICB therapy response.

## Conclusion

Our study identifies *GRN* as a key regulator of both chemo-resistance and immunotherapy resistance in SCLC, acting through both tumor-intrinsic mechanisms and interactions with the TME. The integration of GRN into clinical decision-making could provide a valuable tool for patient stratification, allowing more personalized therapeutic strategies. For *GRN*-low patients, combined chemo-immunotherapy offers significant survival benefits, while for *GRN*-high patients, novel approaches targeting the tumor microenvironment or GRN-driven pathways may be required to overcome resistance. Future validation through in vivo experiments and additional clinical datasets will be critical to fully elucidate the therapeutic potential of GRN, including the genetics, epigenetics, and molecular mechanisms underlying its role in resistance, its downstream effectors, interactions within key signaling pathways, and the interplay between tumor-intrinsic and environmental features. Moreover, studies exploring dynamic fluctuations in *GRN* levels during treatment and its interaction with immune checkpoint pathways and T-cell infiltration are needed to deepen our understanding of GRN’s role in shaping tumor immunogenicity and therapeutic resistance.

## Supplementary Information

Supplementary material 1

Supplementary material 2

Supplementary material 3

Supplementary material 4

## Data Availability

We deposited in vitro RNA-seq data into Gene Expression Omnibus with accession number GSE245123. All other datasets used in the study are publicly available.

## Abbreviations

SCLC: Small cell lung cancer
EP: Etoposide and platinum
ICB: Immune checkpoint blockade
PDX: Patient-derived xenograft
GEMM: Genetically engineered mouse models
CDX: Circulating tumor cells-derived xenograft
TME: Tumor microenvironment
FET: Fisher’s exact test
AM: Alveolar macrophage
TAM: Tumor-associated macrophage
