# Supplementary material for: Transcriptional regulatory network analysis identifies GRN as a key regulator bridging chemotherapy and immunotherapy response in small cell lung cancer
Source: J Hematol Oncol. 2025 Feb 5;18:14. doi: 10.1186/s13045-025-01667-5 (PMC11796135; doi:10.1186/s13045-025-01667-5)
Supplement: Supplementary file 4 — Supplementary material 4 [file 13045_2025_1667_MOESM4_ESM.docx]

**Supplementary Information for**

**Transcriptional regulatory network analysis identifies *GRN* as a key regulator bridging chemotherapy and immunotherapy response in small cell lung cancer**

**Supplementary Methods and Materials**

*Constructing a molecular causal network for SCLC*

We downloaded raw sequencing data in FastQ format for 58 SCLC tumor samples in George et al. study (EGAS00001000925)[1] and 79 samples in Jiang et al. study (GSE60052)[2], and then applied the same following preprocessing procedure to minimize potential batch effects. Sequencing reads were aligned to the human reference genome hg19 using Tophat (v2.1) and read counts per RefGene symbol on the UCSC database were estimated using the htseq-count function in SAMtools. One sample from Jiang et al. (SRR1797252) was removed due to a very low number of mapped reads (less than 5M reads, Supplementary Materials). Messenger RNA abundance of 24088 genes was measured as Transcripts Per Million (TPM). The TPM matrix of 135 samples was log2 transformed followed by quantile normalization. The sex for each sample was inferred based on the expression levels of *RPS4Y1* and *XIST*[3]. The batch effects between two datasets were adjusted using ComBat[4] with sex of each sample as a confounding factor. Finally, a TPM matrix of 135 samples having 24088 gene features were generated.

A molecular causal network for SCLC was constructed using gene expression profiles of 135 primary tumors from the two datasets. A total of 9,301 informative genes with detectable expression levels and large variances across samples were included in the network reconstruction process. The expression of the 9,301 genes was discretized into three states: low, normal, and high level, guided by k-means clustering (k = 3) and biological meaningful cutoff values. The gene expression nodes were then imported into the software suite, Reconstructing Integrative Molecular Bayesian Network (RIMBANet), to construct a biological causal network given the data and priors, as previously described[5]. Briefly, the network reconstruction process searches for a directed acyclic graph (DAG) structure $G$ and associated parameters $\Theta$ that can best explain the given data $D$, $P(G,\Theta|D)$. If the structure $G$ is a DAG, then $P\left( G,\Theta| D \right)$ can be decomposed into a series of sub-structures $P\left( G,\Theta| D \right)=\prod_{i} P(G^{i},\Theta^{i}|D)$. With the structures X→Y, given by $p\left( X\to Y | D \right)=p\left( Y | X,D \right)p(X|D)$, and Y→X, given by $p\left( Y\to X | D \right)=p\left( X | Y,D \right)p(Y|D)$, are no longer equivalent, so that potential causal relationships between X and Y can be inferred unambiguously. To speed up the searching process, for each gene, the bottom 20% genes based on their mutual information were excluded as potential candidate regulators (sparse candidate search[6]). The network reconstruction process is a Monte Carlo Markov chain (MCMC) process. Given different random seeds, we might end up with different structures. Thus, we ran 1000 independent MCMC processes based on 1000 random seed numbers that resulted in 1000 candidate structures. Then, we selected consensus structure features with posterior probabilities >0.3 among candidate structures[7]. Finally, loops in the consensus network were removed by deleting the weakest link in the loops. The resulting network was visualized using Cytoscape3.7[8]. Given a set of seed nodes $N_{s}$, ${SN}_{s}=\bigcup_{i} d(node,N_{s}^{i})\leq l)$ is the union of nodes that are within $l$ steps from the seed node $N_{s}^{i}$, and the subnetwork for the seed nodes $N_{s}$ is the set of connections among $N_{s}$.

Key Driver Analysis (KDA) identified putative master regulators associated each signature set[9]. For each node in the network, we compared neighboring nodes within one or two layers for each signature to obtain a list of potential master regulators whose overlap is statistically significant (FET FDR < 0.05).

*SCLC signatures derived from public datasets*

TPM matrix and model annotation of 19 PDX models of SCLC (GSE110853) were downloaded from the GEO database. In their paper, 359 genes were negatively correlated with EP responses (Spearman coefficient < -0.6)[10] and we labeled them as *EP res*. For genetically engineered mouse models[11], we downloaded DEseq2 results of cisplatin treated (resistant) vs. vehicle (sensitive) mice comparison and filtered genes at FDR<0.01. The mouse genes in the signatures were converted to corresponding human orthologs and the final gene sets consisted of 223 resistant (*Cisplatin res*) and 339 sensitive (*Cisplatin sen*). These signatures are listed in Supplementary Table 1.

*SCLC cell line data*

RNA-seq data of 50 SCLC samples from CCLE dataset[12] were used to investigate tumor intrinsic variation of *GRN*. The RSEM matrix and somatic missense mutation information of the 50 samples were downloaded from CCLE data portal (<https://portals.broadinstitute.org/ccle/data>). RSEM values were log2-transformed and low expressing genes were filtered out (log2(RSEM) < 0.02) and total 24189 genes were collected.

*GRN*-associated genes were identified based on Pearson correlation at FDR<0.01 and separated into *GRN* positive (GRN^+^) and *GRN* negative (GRN^-^) sets according to the direction of the association. To identify enriched functions of the GRN^+^ and GRN^-^ sets were compared with a collection of Hallmark gene sets and GO terms in Molecular Signature Database (MSigDB)[13]. The significance of the overlap with query genes were tested via the Fisher’s Exact Test (FET). The FET p-value was adjusted by Benjamini-Hochberg to measure FDR.

For drug response data[14], we downloaded all data from GDSC download page (<https://www.cancerrxgene.org/downloads/bulk_download>). The microarray (Affymetrix Human Genome U219 Array) profiles of 61 SCLC cells. In addition, IC_50_ values of 396 drugs were downloaded from GDSC1 (304) and GDSC2 (152). If duplicated ones were found, IC_50_ values in GDSC2 dataset superseded corresponding ones in GDSC1 as suggested in their website instruction. Then, we tested GRN expression association with IC_50_ values using Pearson correlation.

*scRNA-seq data analysis*

We utilized three sets of SCLC single-cell RNA-seq data: 1) SCLC tumor cells from *Trp53*^f/f^;*Rb1*^f/f^;*Pten*^f/f^ GEMM (GSE161741)[15], 2) SCLC tumor cells from CDXs (GSE138474)[16] from SCLC patients (SC4, SC16, SC39, SC49, SC53, SC55, SC68, SC53_treated, and SC68_treated), and 3) SCLC primary tumors from 11 patients (PRJCA006026)[17]. For each dataset, Cell Ranger output files are (barcodes.tsv, genes.tsv, and matrix.mtx) downloaded using the accession numbers. Using Seurat package[18], we processed raw reads in a similar way as described in their original papers. Cells with fewer than 200 features and with high proportion of mitochondria genes (mt>10%) were removed. The UMI counts were transformed and normalized by the “NormalizeData” function using the scale factor to 10,000 total UMIs per cell. Then nCount_RNA and percent.mt were further used in the “ScaleData” functions to remove potential bias introduced by batch effect or mitochondrial genes. PCA was performed using the “RunPCA” function using features selected by the Variable Features. Then t-SNE transformation was performed using the “RunTSNE” function using default parameters.

The cancer cells from vehicle and cisplatin-treated murine models were further clustered as described in the paper[15] (resolution=0.1) and two and three clusters from vehicle and treated mouse were determined. For the primary SCLC tumors[17], the representative cell type annotation (cancer, epithelial, T, B, mast, myeloid, and fibroblast) was downloaded using the accession number. We further sub-clustered myeloid cells into alveolar macrophage (AM) 1, 2, and 3, tumor-associated macrophages, monocytes, dendritic cells, and plasmacytoid dendric cells) based on marker genes provided by the paper[17] as well as lung cancer specific myeloid cell markers, for cancer[19]^83^.

The “aREA” function from viper package[20] were used to perform single sample gene set enrichment analysis (ssGSEA) to measure normalized enrichment score (NES) of GRN^+^ and GRN^-^ genes in the single-cells.

*Cell component estimations for SCLC bulk tumors*

ProM (a deconvolution method leveraging both reference profiles and cellular markers derived from scRNA-seq data) was applied to estimate immune, fibroblast and cancer cell proportions in bulk gene expression profiles[21]. The ProM method can further distinguish cell subtypes within major cell types. The primary SCLC scRNA-seq dataset[17] was used as the reference dataset.

*Cell lines*

All cells were cultured at 37°C in a humidified incubator at 5% CO_2_. SCLC cell lines NCI-H2081, NCI-H524 and SHP-77 were maintained in RPMI1640 (Gibco), and supplemented with 10% Fetal Bovine Serum (FBS, Sigma) and 1mM Penicillin Streptomycin (P/S, Gibco). HEK293T cells were maintained in Dulbecco’s Modified Eagle’s Medium (DMEM) and supplemented with 10% FBS (Sigma) and 1mM P/S. Cultured cells were regularly tested for mycoplasma using the mycoAlert Detection Kit (Lonza).

*Western Blot and Antibodies*

Protein lysates were prepared by washing cells with 1 ml of cold PBS and resuspended in lysis buffer (150 mM NaCl, 50 mM Tris-HCl at pH 8.0, 1% NP-40, 0.5% Na deoxycholate, 0.1% SDS, protease inhibitors) for 30 min at 4°C. Lysates were centrifuged at 5°C for 15 minutes at 13,000 rpm to remove insoluble debris. Protein concentrations were quantified using Pierce™ BCA (Thermo Fisher Scientific). Proteins were separated by electrophoresis on a SDS-PAGE gel (BioRad), transferred to a PVDF membrane (Thermo Fisher Scientific) and blocked with 5% milk in Tris-buffered saline with Tween-20 (TBS-T). The membranes were immunoblotted with anti-GRN (18410-1-AP, Proteintech) or anti-vinculin (Sigma).

*Lentiviral introduction of genes*

*GRN* or *GFP* open reading frame (ORF) was cloned into pLEX_307 (a gift from David Root, Addgene #41392) using the Gateway® cloning methods according to manufacturer’s recommendations (Thermo Fisher Scientific). HEK293T cells were seeded in a 10 cm tissue culture dish and incubated at 37°C and 5% CO_2_. At 80% confluency the cells were co-transfected with 10 μg of plasmid constructs, 7.5 μg of psPAX2 (a gift from Didier Trono, Addgene #12260) and 2.5 μg of pMD2.G (a gift from Didier Trono, Addgene #12259) vectors using TransIT-Lenti (Mirus) following manufacturer’s recommendations. At 48h post transfection, virus-containing supernatants were collected, filtered (0.45 μm) and stored at −80°C until use. Cells were infected with lentiviral supernatant supplemented with lentiBlast (OZ BioSciences) at a ratio of 1:1000. Cells were selected with puromycin (1-2 μg/mL for 4-6 days).

*CRISPR-Cas9 genome editing*

Cells with stable human codon-optimized S. pyogenes Cas9 expression were generated by infection with the lentiCas9-Blast plasmid (a gift from Feng Zhang, Addgene # 52962). sgRNAs targeting *GRN* were selected from the Brunello library (*56*). Non-target sgRNA from the Gecko library v2 (*57*) were used as non-target sgRNAs. sgRNA target sequences are listed in Supplementary Table S8. sgRNAs were cloned using BbsI site downstream of the human U6 promoter in a lentiviral vector containing *eGFP* downstream of the human *PGK* promoter (a kind gift of Brown laboratory, ISMMS). Lentivirus was produced as described above. Cas9 expressing cells were then infected with pLenti-GFP-sgRNA.

*Cell viability assay*

Cells were seeded in a 96-well plate at a density of 5,000 cells/well with five replicates. Each well was treated with an equimolar ratio of etoposide (1226, TOCRIS) and cisplatin (2251, TOCRIS) at concentrations ranging from 1nM to 10 µM for 96 hours. Cell viability was measured using alamarBlue Cell Viability Reagent (Thermo Fisher Scientific) and fluorescence at 585nm was measured on a Spectra Max3 plate reader (Molecular Devices, CA) according to the manufacturer’s protocol at excitation of 555 nm. IC50 was calculated by GraphPad Prism (v9) with non-fit model.

*RNA-seq*

Total RNAs from parental and engineered NCI-H2081 cells with either *GFP* or *GRN* were extracted using RNeasy kit (Qiagen). Poly-adenylated RNA was enriched from 1ug of RNA for each sample with the NEBNext® PolyA mRNA Magnetic Isolation Module (NEB), incubated at 94°C for 15 min and double-strand cDNA was synthesized using SuperScript III reverse transcriptase (Thermo Fisher Scientific) and NEBNext® Ultra™ II Directional RNA Second Strand Synthesis Module (NEB). Up to 10 ng of cDNA was used for the Illumina sequencing library construction using NEBNext® Ultra™ DNA Library Prep Kit (NEB). Paired ends sequencing was performed on Novaseq 6000 (Illumina) for 150 nucleotides from each end according to the manufacturer’s instructions. The sequencing reads were aligned to the reference genome (hg19) using the STAR aligner in two-pass mode, and gene read counts were quantified using HTSeq. DESeq2 was used to identify differentially expressed genes between control and GRN-overexpressed H2081 cells. The RNA-seq data is deposited into gene expression omnibus with accession number GSE245123.

**Supplementary Figures**

**
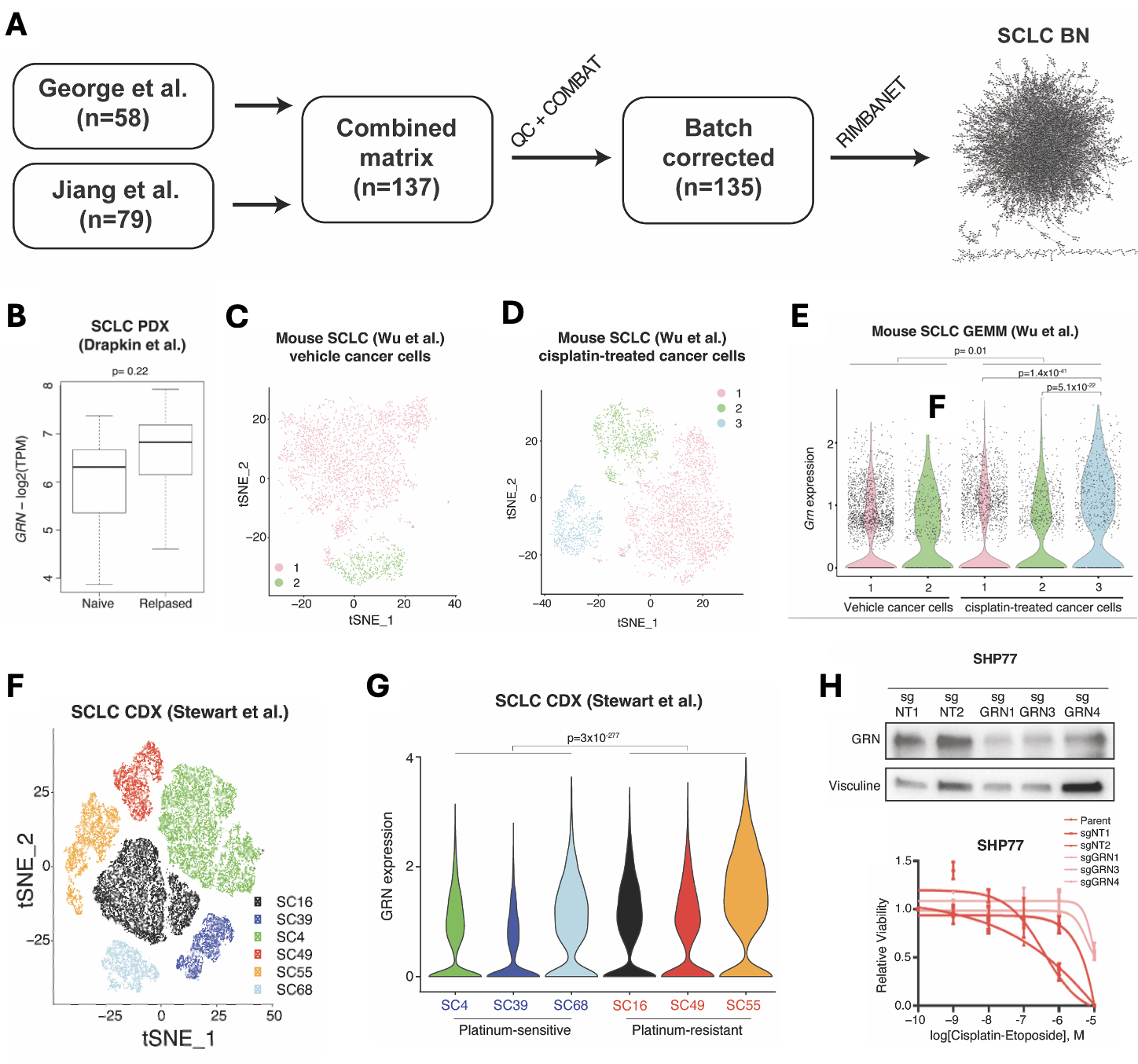
**

**Figure S1. A.** Construction of small cell lung cancer Bayesian networks using two independent SCLC cohorts. To overcome the lack of statistical power for causal inference, two of the largest publicly available SCLC tumor transcriptomic datasets (58 and 79 tumors from Germany[1] and China[2], respectively) were combined into one transcriptomic dataset consisting of 135 samples after adjusting for batch effect. Then, a Bayesian Network (BN) was constructed via RIMBANET algorithm[5] to derive causal relationships among genes; the resulting SCLC network consist of 8451 unique genes (nodes) connected via 9301 regulations (edges). **B.** *GRN* expression between treatment naïve and relapsed PDX samples. *GRN* expression was also numerically higher in relapsed PDX samples compared to treatment naïve PDXs while not statistically significant. **C-D.** T-SNE plot of cancer cells from vehicle (**C**) and cisplatin-treated (**D**) *Rb1*^f/f^;*Trp53*^f/f^;*Pten*^f/f^ (RPP) GEMM. Two and three clusters within the treatment groups are annotated in the original paper[15]. Cisplatin treatment induced a unique EMT-associated non-NE SCLC cell population (cluster 3) with acquired resistance through cancer cell evolution during the treatment[15]. **E.** *GRN* expression (y-axis) in cancer cells from vehicle- and cisplatin-treated animals. *Grn* expression was only marginally higher in cisplatin-treated cancer cells compared to vehicle controls (p-value= 0.01), mostly due to the two common cells clusters (clusters 1 and 2 in both vehicle and cisplatin-treated. By contrast, *Grn* was significantly higher in the cluster 3 in the cisplatin-treated cells (p-value= 1.4×10^-41^ and 5.1×10^-22^ for comparison with the clusters 1 and 2, respectively). **F.** T-SNE plot of 6 Circulating Tumor Cells (CTC) derived xenograft (CDX) models with distinct chemo-sensitivity[16]. **G.** *GRN* expression (y-axis) in cisplatin-sensitive (SC39, SC4, and SC68) and -resistant (SC16, SC49, and SC55) CDX models[16]. *GRN* expression were significantly higher in chemo-resistant samples compared to sensitive ones (p-value= 3.4×10^-277^). **H.** (Above) Western blots confirming GRN knockdown in SHP77 cells. (Bottom) Viability of SHP77 with equimolar treatment of EP for 96-hour relative to vehicle control. No differences are observed.

**
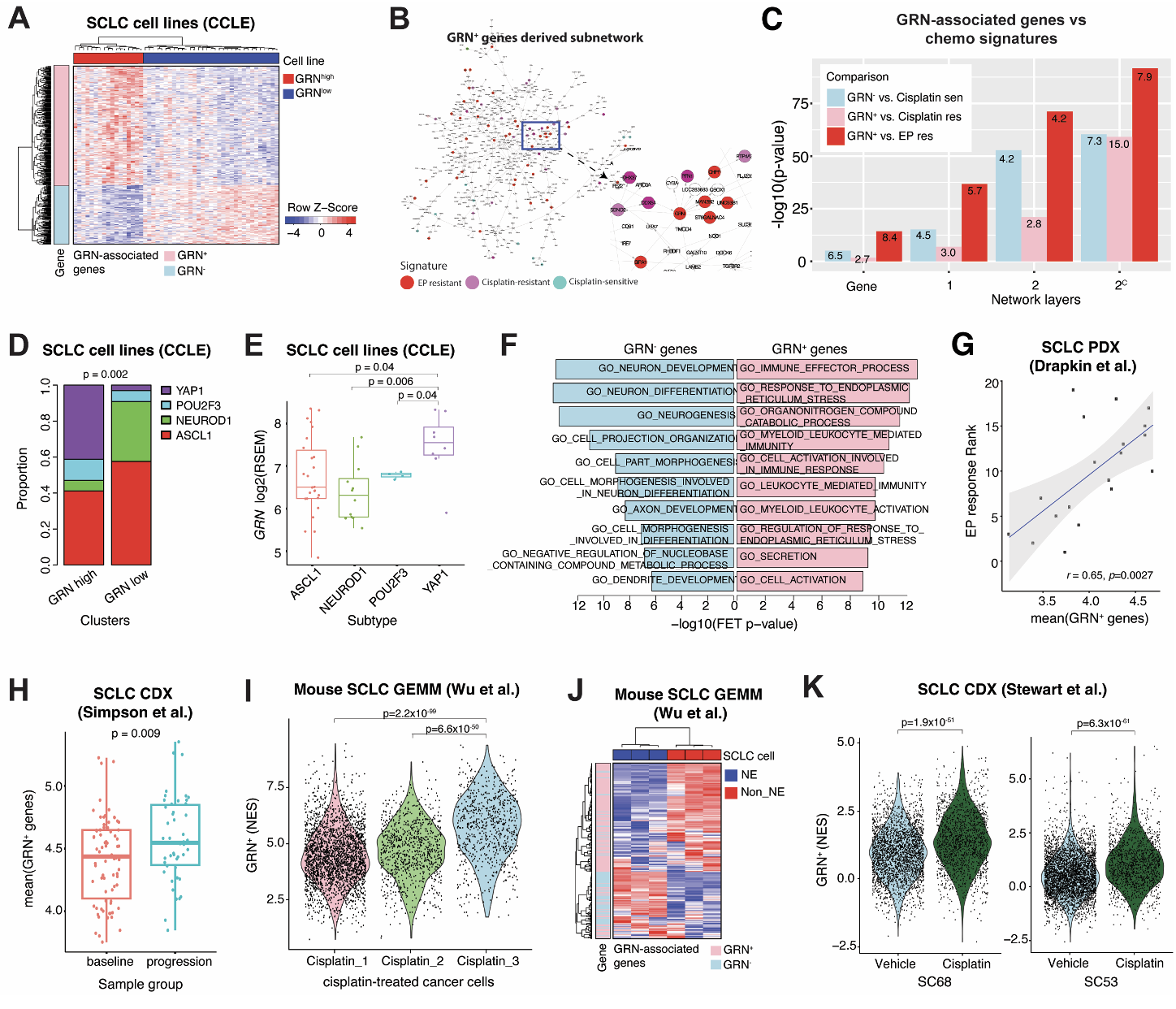
Figure S2. A.** Hierarchical clustering of 50 SCLC cells into two groups using *GRN*-associated genes (249 GRN^+^ and 124 GRN^-^ genes). **B**. The largest connected subnetwork of two-layer neighboring nodes using GRN^+^ genes as seeds. *GRN*-associated genes in cell lines were tightly connected in the SCLC network constructed based on gene expression of human tumor tissues. **C.** Overlaps of *GRN*-associated genes and their network neighboring nodes with chemo-response signature genes (GRN^+^ vs. EP res/Cisplatin res and GRN^-^ vs Cisplatin sen); Gene: direct overlap without network expansion, 1: overlap of one-layer neighboring nodes from seeds, 2: overlap of two-layer neighboring nodes from seeds, 2^c^: the largest connected subnetwork of two-layer neighboring nodes from seeds. The number on each bin indicates OR of overlapping genes. FET p-values (y-axis) are -log10 transformed. **D**. Proportion of SCLC molecular subtypes (y-axis) in GRN^high^ and GRN^low^ groups. Hierarchical clustering based on the *GRN*-associated genes separated the 50 SCLC cell lines into two clusters: 17 GRN^high^ cell lines and 33 GRN^low^ cell lines. Among the SCLC subtypes previously defined by the four lineage transcription factors (ASCL1, NEUROD1, POU2F3, and YAP1)[22], the two clusters are significantly associated with SCLC subtypes (Chi-square test p= 0.002). **E**. *GRN* expression (y-axis) within SCLC subtypes determined by expression of the four-lineage transcription factors (ASCL1, NEUROD1, POU2F3, and YAP1)[22]. *GRN* expression is significantly higher in SCLC-Y than in SCLC-A and SCLC-N subtypes (p-value= 0.043 and 0.006, respectively. P-values of two-sided t-test are calculated to assess *GRN* expression differences. **F.** Top 10 enriched gene ontology (GO) terms among *GRN*-associated genes. FET p-values (x-axis) are -log10 transformed. **G**. Correlation of average expression levels of the GRN^+^ genes (x-axis) and the EP response rank of 19 PDX models (y-axis). Pearson correlation coefficients and p-value are calculated to assess the association. **H**. Average GRN^+^ expression levels (y-axis) in baseline and progressed tumors from SCLC CDX. Two-sided t-test p-value is calculated to assess the difference. **I**. Classification of NE and non-NE cancer cells from RPP GEMM based on *GRN*-associated genes. **J.** Normalized enrichment scores (NES) of the GRN^+^ genes (y-axis) in SCLC cells in the three clusters from cisplatin-treated mouse. **K.** NESs of the GRN^+^ genes (y-axis) in SCLC cells from vehicle- and cisplatin-treated CDX models. (**J**-**K**) Expression differences are assessed by Wilcoxon rank-sum test.


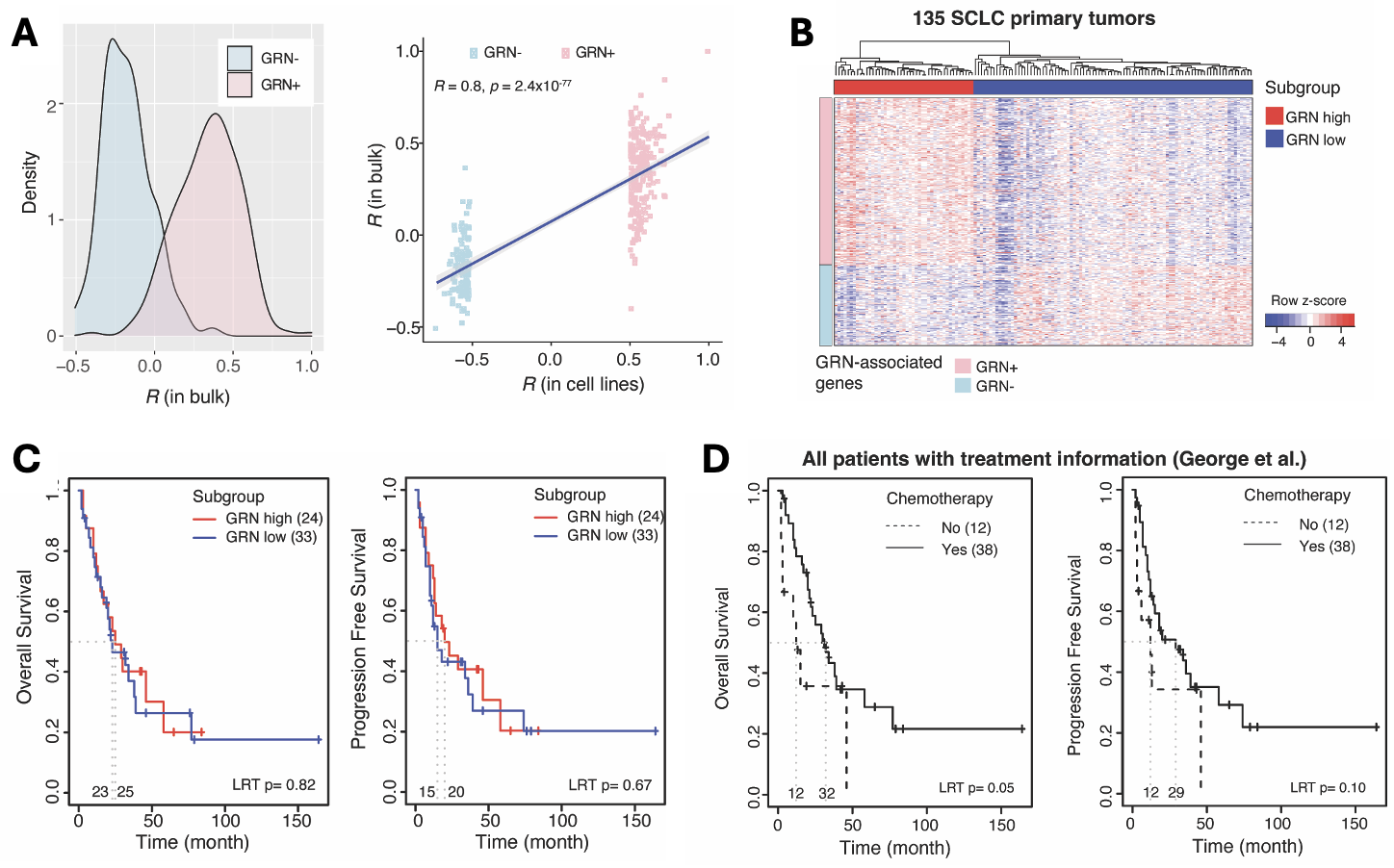


**Figure S3. A.** Given that cancer cell intrinsic variation of *GRN* expression was associated with chemo-resistance, we first sought to test whether the *GRN*-associated genes derived from the cancer cell lines were consistent in the primary SCLC tumors. Distribution of correlation coefficients of *GRN*-associated genes (GRN^+^ and GRN^-^) with *GRN* in bulk tumors (left). Comparison of correlation coefficients in cell lines (x-axis) and bulk tumors (y-axis) (right). About a half of the GRN^+^ and GRN^-^ genes were significantly correlated with *GRN* in the 135 primary tumors (at p-value<0.01: 146 out of 227 GRN^+^ and 46 out of 109 GRN^-^ genes). **B.** Hierarchical clustering of the 135 tumors into two distinct groups based on *GRN*-associated genes. Using all cancer cell intrinsic GRN^+^ and GRN^-^ genes, we classified primary tumors into two groups: one group consisting of 45 samples with high expression of GRN^+^ and low expression of GRN^-^ genes (*GRN*-high) and the other group of 90 samples with opposite patterns (*GRN*-low). **C**. KM plots (left: OS and right: PFS) showing no survival differences between *GRN*-high and *GRN*-low groups. Log rank test p-values are calculated to assess survival differences between the groups. **D**. KM plots (left: OS and right: PFS) showing survival differences between chemo-treated and not-treated patients without patient classification. Log rank test (LRT) p-values are calculated to assess survival differences between the groups.

**
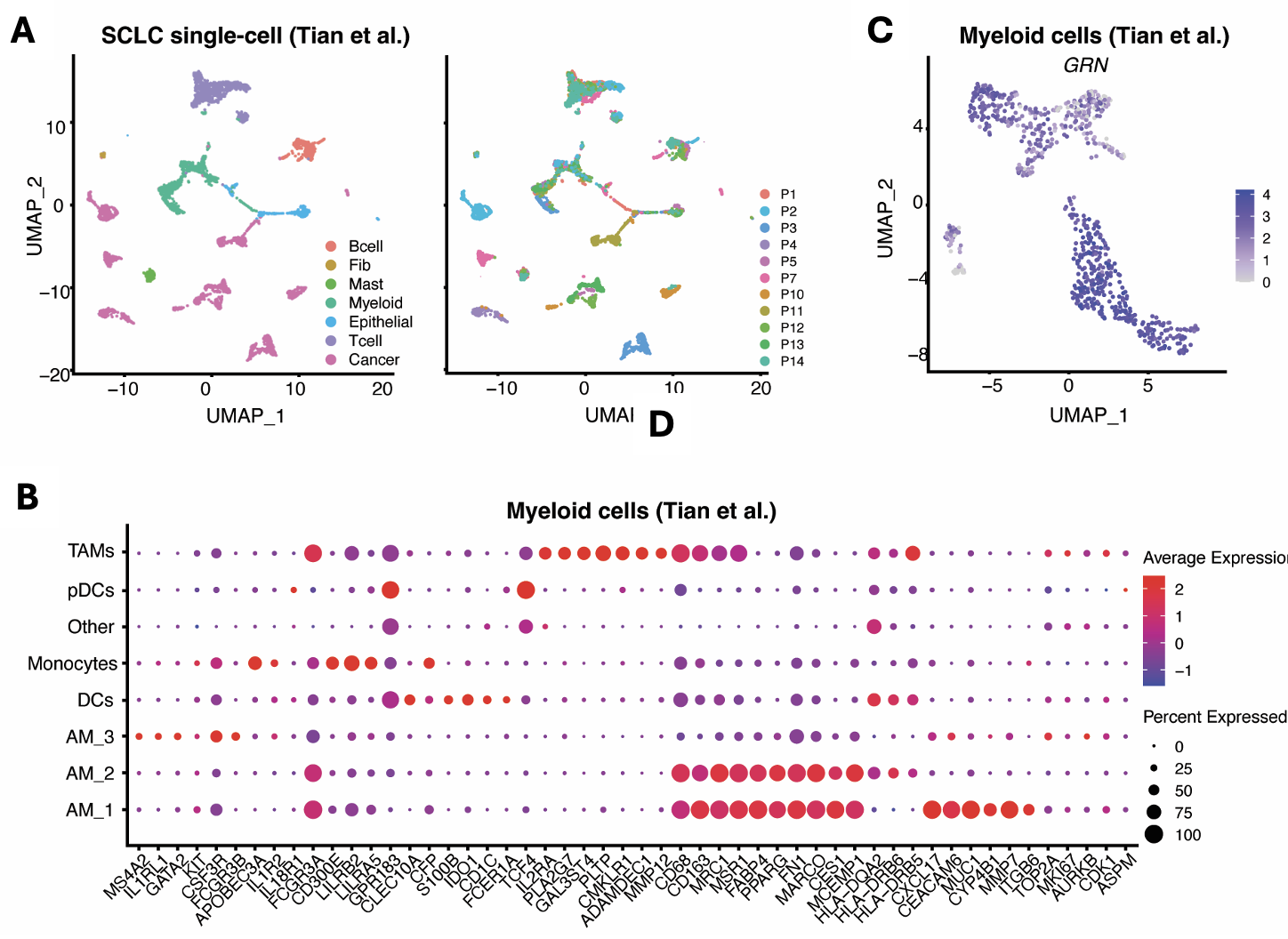
**

**Figure S4. A.** To further understand cellular source of *GRN* in SCLC tumors, we investigated a recently published single-cell transcriptomic data of lung tissues from 11 patients with limited-stage SCLC[17]. UMAP plot showing clusters of cells in different cell types (left) and individuals (right). Among various cell types in the lung tissues from SCLC tumors, cancer cells showed prominent heterogeneity across patients, but immune, stromal, or normal epithelial cells from different patients largely clustered together. **B.** Bubble plot showing expression of marker genes for myeloid subtypes. We further annotated myeloid cells based on known myeloid cell subtype markers[17]. **C.** Expression of *GRN* in myeloid cells from SCLC tumors.

**REFERENCES:**

1. George, J., et al., *Comprehensive genomic profiles of small cell lung cancer.* Nature, 2015. **524**(7563): p. 47-53.

2. Jiang, L., et al., *Genomic Landscape Survey Identifies SRSF1 as a Key Oncodriver in Small Cell Lung Cancer.* PLoS Genet, 2016. **12**(4): p. e1005895.

3. Yoo, S., et al., *MODMatcher: multi-omics data matcher for integrative genomic analysis.* PLoS Comput Biol, 2014. **10**(8): p. e1003790.

4. Leek, J.T., et al., *The sva package for removing batch effects and other unwanted variation in high-throughput experiments.* Bioinformatics, 2012. **28**(6): p. 882-3.

5. Zhu, J., et al., *Integrating large-scale functional genomic data to dissect the complexity of yeast regulatory networks.* Nat Genet, 2008. **40**(7): p. 854-61.

6. Friedman, N., et al., *Using Bayesian networks to analyze expression data.* J Comput Biol, 2000. **7**(3-4): p. 601-20.

7. Zhu, J., et al., *Increasing the power to detect causal associations by combining genotypic and expression data in segregating populations.* PLoS Comput Biol, 2007. **3**(4): p. e69.

8. Su, G., et al., *Biological network exploration with Cytoscape 3.* Curr Protoc Bioinformatics, 2014. **47**: p. 8 13 1-24.

9. Yoo, S., et al., *Integrative network analysis of early-stage lung adenocarcinoma identifies aurora kinase inhibition as interceptor of invasion and progression.* Nat Commun, 2022. **13**(1): p. 1592.

10. Drapkin, B.J., et al., *Genomic and Functional Fidelity of Small Cell Lung Cancer Patient-Derived Xenografts.* Cancer Discov, 2018. **8**(5): p. 600-615.

11. Bottger, F., et al., *Tumor Heterogeneity Underlies Differential Cisplatin Sensitivity in Mouse Models of Small-Cell Lung Cancer.* Cell Rep, 2019. **27**(11): p. 3345-3358 e4.

12. Ghandi, M., et al., *Next-generation characterization of the Cancer Cell Line Encyclopedia.* Nature, 2019. **569**(7757): p. 503-508.

13. Liberzon, A., et al., *The Molecular Signatures Database (MSigDB) hallmark gene set collection.* Cell Syst, 2015. **1**(6): p. 417-425.

14. Iorio, F., et al., *A Landscape of Pharmacogenomic Interactions in Cancer.* Cell, 2016. **166**(3): p. 740-754.

15. Wu, Q., et al., *YAP drives fate conversion and chemoresistance of small cell lung cancer.* Sci Adv, 2021. **7**(40): p. eabg1850.

16. Stewart, C.A., et al., *Single-cell analyses reveal increased intratumoral heterogeneity after the onset of therapy resistance in small-cell lung cancer.* Nat Cancer, 2020. **1**: p. 423-436.

17. Tian, Y., et al., *Single-cell transcriptomic profiling reveals the tumor heterogeneity of small-cell lung cancer.* Signal Transduct Target Ther, 2022. **7**(1): p. 346.

18. Butler, A., et al., *Integrating single-cell transcriptomic data across different conditions, technologies, and species.* Nat Biotechnol, 2018. **36**(5): p. 411-420.

19. Bischoff, P., et al., *Single-cell RNA sequencing reveals distinct tumor microenvironmental patterns in lung adenocarcinoma.* Oncogene, 2021. **40**(50): p. 6748-6758.

20. Alvarez, M.J., et al., *Functional characterization of somatic mutations in cancer using network-based inference of protein activity.* Nat Genet, 2016. **48**(8): p. 838-47.

21. Wang, L., et al., *Single-cell transcriptomic-informed deconvolution of bulk data identifies immune checkpoint blockade resistance in urothelial cancer.* iScience, 2024. **27**(6): p. 109928.

22. Patel, A.S., et al., *Prototypical oncogene family Myc defines unappreciated distinct lineage states of small cell lung cancer.* Sci Adv, 2021. **7**(5).
